# Machine Learning Based Automated Segmentation and Hybrid Feature Analysis for Diabetic Retinopathy Classification Using Fundus Image

**DOI:** 10.3390/e22050567

**Published:** 2020-05-19

**Authors:** Aqib Ali, Salman Qadri, Wali Khan Mashwani, Wiyada Kumam, Poom Kumam, Samreen Naeem, Atila Goktas, Farrukh Jamal, Christophe Chesneau, Sania Anam, Muhammad Sulaiman

**Affiliations:** 1 Department of Computer Science & IT, The Islamia University of Bahawalpur, Bahawalpur 61300, Pakistan; aqibcsit@gmail.com (A.A.); salman.qadri@iub.edu.pk (S.Q.); samreencsit@gmail.com (S.N.); 2 Institute of Numerical Sciences, Kohat University of Sciences & Technology, Kohat 26000, Pakistan; mashwanigr8@gmail.com; 3 Program in Applied Statistics, Department of Mathematics and Computer Science, Faculty of Science and Technology, Rajamangala University of Technology Thanyaburi (RMUTT), Thanyaburi, Pathumthani 12110, Thailand; 4 Center of Excellence in Theoretical and Computational Science (TaCS-CoE) & KMUTT Fixed Point Research Laboratory, Room SCL 802 Fixed Point Laboratory, Science Laboratory Building, Departments of Mathematics, Faculty of Science, King Mongkut’s University of Technology Thonburi (KMUTT), 126 Pracha-Uthit Road, Bang Mod, Thrung Khru, Bangkok 10140, Thailand; 5 Department of Medical Research, China Medical University Hospital, Taichung 40402, Taiwan; 6 Department of Statistics, Mugla Sıtkı Koçman University, Mugla 48000, Turkey; gatilla@mu.edu.tr; 7 Department of Statistics, Govt S.A Post Graduate College Dera Nawab Sahib, Bahawalpur 63351, Pakistan; drfarrukh1982@gmail.com; 8 Department of Mathematics, Université de Caen, LMNO, Campus II, Science 3, 14032 Caen, France; christophe.chesneau@unicaen.fr; 9 Department of Computer Science, Govt Degree College for Women Ahmadpur East, Bahawalpur 63350, Pakistan; sania.anam7@gmail.com; 10 Department of Mathematics, Abdul Wali Khan University Mardan, Mardan 23200, Pakistan; sulaiman513@yahoo.co.uk

**Keywords:** diabetic retinopathy, clustering, segmentation, hybrid features, classification

## Abstract

The object of this study was to demonstrate the ability of machine learning (ML) methods for the segmentation and classification of diabetic retinopathy (DR). Two-dimensional (2D) retinal fundus (RF) images were used. The datasets of DR—that is, the mild, moderate, non-proliferative, proliferative, and normal human eye ones—were acquired from 500 patients at Bahawal Victoria Hospital (BVH), Bahawalpur, Pakistan. Five hundred RF datasets (sized 256 × 256) for each DR stage and a total of 2500 (500 × 5) datasets of the five DR stages were acquired. This research introduces the novel clustering-based automated region growing framework. For texture analysis, four types of features—histogram (H), wavelet (W), co-occurrence matrix (COM) and run-length matrix (RLM)—were extracted, and various ML classifiers were employed, achieving 77.67%, 80%, 89.87%, and 96.33% classification accuracies, respectively. To improve classification accuracy, a fused hybrid-feature dataset was generated by applying the data fusion approach. From each image, 245 pieces of hybrid feature data (H, W, COM, and RLM) were observed, while 13 optimized features were selected after applying four different feature selection techniques, namely Fisher, correlation-based feature selection, mutual information, and probability of error plus average correlation. Five ML classifiers named sequential minimal optimization (SMO), logistic (Lg), multi-layer perceptron (MLP), logistic model tree (LMT), and simple logistic (SLg) were deployed on selected optimized features (using 10-fold cross-validation), and they showed considerably high classification accuracies of 98.53%, 99%, 99.66%, 99.73%, and 99.73%, respectively.

## 1. Introduction

Diabetic retinopathy (DR) is one of the severe dysfunctions of the human eye [1]. If a person has diabetes for a long time, along with an uncontrolled blood sugar level, he/she is at a clearly high risk of having visual complications. DR takes years to progress, and a well-controlled blood sugar level can slow down DR progression [2]. Additionally, DR is one of the foremost causes of sightlessness, and its time taking progression makes it curable if diagnosed in early stage; however, in the case of failure in its early stage detection, it can damage the human eye’s retina, leading to uncurable sightlessness [3]. The normal retinal assessment of diabetic patients is useful for the early identification of DR, considerably reducing blindness occurrence. However, retinal screening is time-consuming and requires highly qualified ophthalmologists to scrutinize the fundus photographs probing the retinal lesions [2].

It has been shown that DR sometimes occurs due to characteristic changes in the retina, like diametric change in the blood vessels (which are made up of light-sensitive tissues) that lie near the optical nerve [4]. Light rays coming into the eyes are collected on the retina, and the transmission of vision signal to the brain is made through optical nerves where interpretation is done [4]. Macula (the small surface area at the midpoint of retina) is responsible for the recognition of face and read–write functions. The peripheral retina is a part of the retina surrounding macula that is responsible for effective communication between brain and eye [5].

Non-proliferative (NP) DR is a stage of DR where human blood vessels in the retina get weak, and very small dot sized hemorrhages are formed after the swelling of blood vessels; this swelling and hemorrhaging causes an edema in the retina which affects vision with mild symptoms [6]. If NP-DR is not treated effectively, it becomes proliferative (P) DR—an advanced stage of DR where a major component of human vision—the retina—gets oxygen deprived, leading to neovascularization, which results in the formation of spots or floaters; these are reasons for the loss of vision [6].

Dots, spots, and cobweb-like strings floating in the patient’s eye are the main symptoms of DR that are recognized by researchers [7]. Sometimes, a periodic change in sightedness from clear to blurry vision is observed with time. In some patients, dark/black areas are identified in the eye; these cause poor vision in low light or night, which leads to a complete loss of vision [7]. In some cases, vision loss is due to optic disc extraction, which is now becoming a serious complication of DR [8].

In our modern society, with the advancement of science and technology, humans have faced many diseases associated with age. In particular, the human eye can be divided into two classes: The first one is the dysfunctional eye due to illnesses like glaucoma, blepharitis, and conjunctivitis [9]. The other class of human eye diseases comprise the malfunctioning of the human eye due to other life-related diseases like hypertension, diabetes, and arteriosclerosis. Diabetes affects the human eye by damaging blood vessels in eye retina, causing symptoms from minor vision problems to complete vision loss—this is called DR [9]. The early diagnosis and treatment of DR help protect the eye form sever stages; these are done via retinal images called fundus images (FI), which are captured by using a medical image camera, and the disease is detected by a highly expert ophthalmologist [6]. For diseases falling in first-class mentioned above, assessment is done by the quantitative measurement of retinal deviation, but this class assessment is difficult in early stages because it is related to drusens and normally appears as a form of black/dark/yellow drops or cobweb-like strings in the eye [7]. In modern word color, retinal images (RIs) are used for the detection of drusens that accrue in the retina. Automated detection and segmentation have thus far prominently contributed to the provision of essential information for identifying the stages of DR [4].

### 1.1. Literature Review

Pires, R. et al. extracted information from retinal images. Neural networks were applied in this regard. This research extracted features that analyzed the patient and tried to find imperfections. The suggested technique attained a receiver–operating characteristic (ROC) area under the curve of 98.2%. The given technique was applied to the Messidor-2 dataset. Similar results obtained by using two-to-five-fold cross-validation protocols when applied to the Messidor-2 and DR2 datasets. Upon referral assessment, this method achieved the highest result, which was 99.0% when convolutional neural networks (CNNs) were used [10]. Zhang, W. et al. described an automatic DR identification system and a classification system. The system identified the occurrence and harshness of DR using fundus photographs. Additionally, an NN was applied. In this research, a high-class dataset was obtained from images. The identification model achieved the best result, with 97.5% sensitivity, 97.7% specificity, and 97.7% area under the curve. The classification model achieved 98.1% sensitivity and 98.9% specificity [11]. Harun, N. H. et al. classified fundus images. For this purpose, a multi-layer perceptron (MLP) ANN employed. The purposed system was trained for classification using Levenberg–Marquardt (LM) and Bayesian regularization (BR), and 19 extracted features were used in the NN to classify images. The MLP trained with the BR had a more improved classification rate than that with the LM. The MLP achieved a 72.11% rate for training and a 67.47% rate for testing with the BR [12]. Verbraak, F. D. et al. determined a system that automatically detected diabetic retinopathy. A total of 1616 people (having diabetes) were imaged. RIs were classified using hybrid deep learning enhanced devices and were also evaluated with a reference standard. The hybrid deep learning enhanced device achieved 99% sensitivity and 97.8% specificity, with respect to the reference standard for vision threatening (vt) DR, while the more than mild (mtm) DR device achieved 79.4% sensitivity and 93.8% specificity with respect to the reference standard. This proved that hybrid deep learning enhanced the device and achieved a high accuracy for the detection of vtDR and mtmDR [13]. Afrin, R. and Shill, P. C. proposed an automatic system for detecting retinal wounds using RIs, and they also categorized the phases of DR. The knowledge-based fuzzy classifier was applied to extracted features for classification, achieving 95.63% accuracy for the classification [14]. Parmar, R. et al. detected DR by using RIs. In this research, images were classified into five classes. An NN was applied in this model. This model achieved 85% accuracy [15]. Xu, K. et al. used an NN method for categorization of DR through the use of color FIs. The suggested method achieved 94.5% accuracy [16]. Sim, D. A. et al. proposed a system that used automatic real-time calculation for further analysis and medical appointment, if necessary. Moreover, the real-time calculation was combined with microelectronic medicinal records to enable more precise predictions for specific patients. In particular, a 7.1% accuracy of mentionable infection was achieved, 97.4–99.1% accuracy was achieved for sensitivity, and 98.3–99.3% accuracy was achieved for specificity [17]. Gulshan, V. et al., described the automatic recognition of DR and diabetic macular edema in RF images. This work reached 97.5% sensitivity and 93.4% specificity on the EyePACS-1 dataset, and it achieved 96.10% sensitivity and 93.90% specificity on the Messidor-2 dataset using deep learning algorithms [18]. Gargeya, R. and Leng, T. described strong analytical expertise for automatic DR screening. The suggested model attained an area under the curve (AUC) of 0.97 with 94% sensitivity and 98% specificity thought “five-fold cross-validation” on the local dataset. The Messidor-2 database attained a 0.94 AUC, and the E-Ophtha database attained a 0.95 AUC using a data-driven deep learning algorithm [19].

### 1.2. Contribution

The contribution of this study can be summarized as follows:

A novel segmentation technique, which is called clustering-based automated region growing segmentation (CARGS) and includes post optimal feature selection, is introduced. This technique includes four steps.

1. Firstly, the RF image is divided into four equal regions. The group of neighboring seeds is used for the formulation of an identifiable region. These seeds are in the shape of an irregular polygon with a variable radius from the center of an image in order to ensure the maximum chance of grouping seeds that belong to the same region. This allows for any possible size, dimension, and shape to be considered as regions of interest (ROIs). At the post-processing stage, the K-mean algorithm is employed on improved segmented regions.
2. After segmentation, hybrid-features data are extracted.
3. Four machine learning (ML) feature selection classifiers are employed for the post optimal hybrid feature selection.
4. The post-optimized hybrid features dataset is deployed for five ML classifiers, and efficient classification accuracy is acquired.

## 2. Materials and Methods

**Figure 1:**
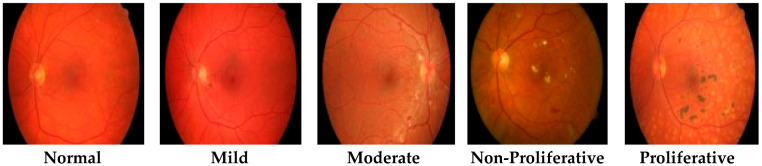
Typical retinal fundus images.

This study comprised a dataset that held five types of RF images; one was that of normal patient retinas and the other four that were DR patient stages named mild, moderate, non-proliferative, and proliferative, as shown in Figure 1. This dataset was collected via a fundus photography machine (Vision Star, 24.1 MegaPixel Nikon D5200 camera) available in Bahawal Victoria Hospital (BVH) Bahawalpur [20], Pakistan. RF images provide better structural changes in blood veins and tissues, and they also provide better contrast enhancement and sensitivity. For each type, a hundred RF images sized 256 × 256 and a total of 2500 (500 × 5) RF dataset images of normal and DR patients were acquired. All the images were manually examined by an expert ophthalmologist in light of different medical tests and biopsy reports. Finally, in the presence of a gold standard/ground truth RF image dataset, we proposed a novel CARGS approach.

### 2.1. Proposed Methodology

**Figure 2:**
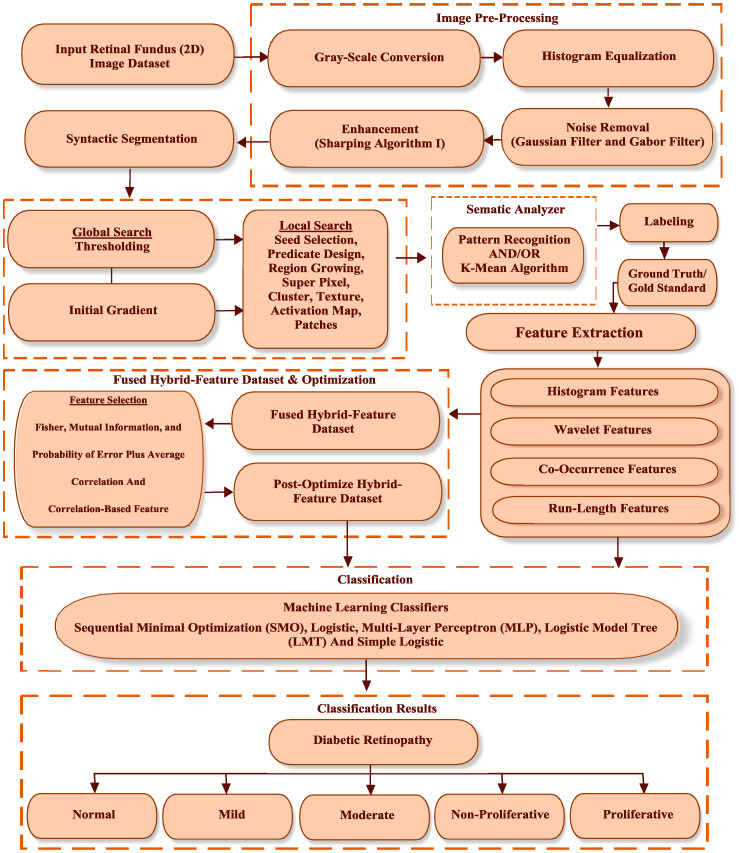
Clustering-based segmentation and hybrid-feature analysis for diabetic retinopathy (DR) classification framework.

The proposed methodology is described in Figure 2. First of all, the proposed algorithm is described with all procedural steps.
***Proposed Algorithm*****Begin** **Main** { **Input**
*ϵ* Retinal fundus image dataset **For** { Steps 1–10Fundus image datasets *ϵ* five retinal types.Image pre-processing.Clustering-based automated region growing segmentation (CARGS).Extract texture features *ϵ* histogram, co-occurrence matrix, run length matrix, and wavelet.Generate fused hybrid-feature dataset.Pre-optimization (Fisher (F), probability of error (POE) plus average correlation (AC), and mutual information (MI)) feature selection technique employed on fused hybrid-feature dataset.Extract 30 pre-optimized fused hybrid-feature dataset.Post-optimization (correlation-based feature selection—CFS) feature selection technique and employed pre-optimized fused hybrid-feature dataset.Extract 13 post-optimized, fused hybrid-feature dataset. **End For** }10.ML classifiers are employed on post-optimized, fused hybrid-feature dataset.**Output** = DR classification results **End main** }

Now, let us discuss the proposed methodology in detail. The first step consists of a collection of image datasets, which, in this step, are the RF images of a normal patient and four different types of DR patients, which, for this study, were collected from Bahawal Victoria Hospital Bahawalpur, Pakistan [20]. The second step is image pre-processing. In this step, digital color retinal images are converted into a gray level 8-bit image format. Secondly, noise removal is performed by using “Gaussian and Gabor filters”. Finally, data cleaning is done for the RF image datasets standardization. The third step is segmentation, which helps to remove the extra object and nominates the exact position and refines the texture of the lesion. The fourth step is hybrid feature extraction. In this step, four types of features—histograms, co-occurrence matrixes, run lengths matrix, and wavelet features—are extracted from a standardized RF image dataset. The fifth step is the formation of a fused hybrid-feature dataset using a data fusion technique, which is a very powerful technique for merging multiple features to produce an accurate classification compared to individual features. The sixth step is fused hybrid feature optimization. In this step, we select the best attribute of the extracted hybrid feature dataset. Firstly, 30 pre-optimized hybrid features are selected using ML (F, PA, and MI) feature selection techniques. Secondly, the (CFS) ML feature selection technique is deployed on a pre-optimized dataset, and 13 post-optimized fused hybrid features that are most useful for the classification of DR are selected. The last step is classification, where five ML classifier are employed (using 10-fold cross-validation) on the selected, post-optimized fused hybrid features datasets. Cross-validation, a standard evaluation approach, is a systematic way of obtaining repeated percentages. It follows the following scheme. Break a dataset into 10 parts (“folds”), then hold each part to test, and train the remaining nine at the same time; this returns an average of 10 evaluation results. By initializing, in “validated” cross-validation, it is ensured that there is, approximately, the correct proportion of class values in each class. After 10 times of cross-validation and the counting of the evaluation results, ML requires the learning algorithm to be used one last (11th) time in the final dataset to obtain the model it publishes. These ML classifiers are named sequential minimal optimization (SMO), logistic (Lg), multi-layer perceptron (MLP), logistic model tree (LMT), and simple logistic (SLg).

### 2.2. Image Preprocessing

The acquired (2D) color digital retinal fundus image dataset is converted into gray level (eight-bit) image format, and histogram equalization is used to normalize non-uniformities and improve contrast. The gray level image has a 256 gray-level (0–255), and, in a histogram, the vertical axis rests on a numeral of pixel in the image and the horizontal axis extends from 0 to 255 [21]. The “probability density function” (p.d.f.) of pixel intensity level ${𝓇}_{i}$ is shown in Equation (1):

(1) $$P_{𝓇}\left({𝓇}_{i}\right)=K_{i}/K$$

where $0\;\leq {𝓇}_{i}\leq 1$, $K_{i}$ is the number of pixels with intensity ${𝓇}_{i}$, K represent the total number of pixels and *i* = 0–255.

During the acquisition of RF image data, freckled noise is detected due to the environmental conditions of the image sensor. A noise removal process is adopted to solve this problem. In this process, two image processing filters are used. Firstly, a Gaussian filter [22] is implemented to overcome this issue and enhance the contrast. This filter is expressed below.

Let, Y be a random variable following the Gaussian distribution with a zero mean and standard deviation (S.D) = λ, i.e., $Y\;\sim N\left(0,\lambda^{2}\right)$. In the unidimensional case, the p.d.f. of Y is shown in Equation (2):

(2) f(y)=[1λ. 2π] e− y22λ2

Let, Z be a random variable following the Gaussian distribution with zero mean and S.D = λ, i.e., $Z\;\sim \;N\left(0,\lambda^{2}\right)$. For the multidimensional case, the joint p.d.f. of (Y, Z) is denoted by ψ(y,z), and for the two-dimensional case, it is defined in Equation (3):

(3) ψ(y,z)=[12λ2 π ] e− ( y2+z2)2 λ2

Secondly, the Gabor filter [23] is implemented to enhance and detect edges that are based on the Gaussian kernel function. Mathematically, the Gabor filter is expressed in Equation (4):

(4) $$g\left(y,z,\lambda ,\theta ,\eta ,\sigma ,\gamma \right)=e^{\left(-\frac{\overset{´}{y^{2}}+\gamma^{2}{\overset{´}{z}}^{2}}{2\sigma^{2}}\right)}\;e^{\left(i\;\left(2\pi \frac{\overset{´}{y}}{\lambda }+\eta \right)\right)}$$

In this equation, λ denotes the wavelength of the sinusoidal factor, θ denotes the angle of the normal to the parallel stripes of a Gabor function, η is the point offset, σ is the sigma standard deviation of the Gaussian envelope, and γ is the spatial aspect ratio that specifies the ellipticity of the support of the Gabor filter.

(5) $$\overset{´}{y}=\;\left(y\;cos\theta +z\;sin\theta \;\right)$$

(6) $$\overset{´}{z}=\;\left(-y\;sin\theta +z\;cos\theta \;\right)$$

Here, (y, z) is the image pixel, the scale is a location parameter, θ positioning angle, θ=(kπ)n, (k = 0 to n − 1), where n represents the angles (k = 0 to 7) and 5 location parameters (scale = 1 to 5). By using these techniques, the noisy values are replaced with average values in the image, and a smooth and enhanced RF image dataset can be acquired.

### 2.3. Clustering-Based Automated Region Growing Segmentation (CARGS)

**Figure 3:**
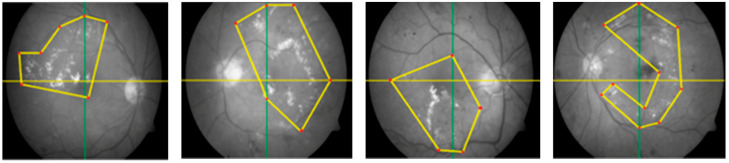
Irregular polygonal seed selection proposed novel mechanism.

Segmentation is a procedure that helps to eliminate the extra object, refines the texture of the lesion, and recommends a precise position. ROI extraction has many automated and semi-automated methods. We know that automated ROI extraction is usually based on the idea of image segmentation, but there is no single method for ideal segmentation. On the other hand, there are semi-automated techniques based on expert opinion, but human-based extraction has some limitations. To solve this problem, CARGS is employed on DR images, which helps to examine the qualitative nature of data and useful information. This idea uses a group of neighboring seeds for the formulation of an identifiable region, which is entirely different approach than using contagious and connected sets of seeds fixed dimensions of 2 × 2, 3 × 3, 4 × 4, 5 × 5, or 8 × 8. These seeds are in the shapes of irregular polygons with variable radii from the center of their image. This methodology of seed selection ensures the maximum chance of grouping seeds that belong to same region, as shown in Figure 3.

**Figure 4:**
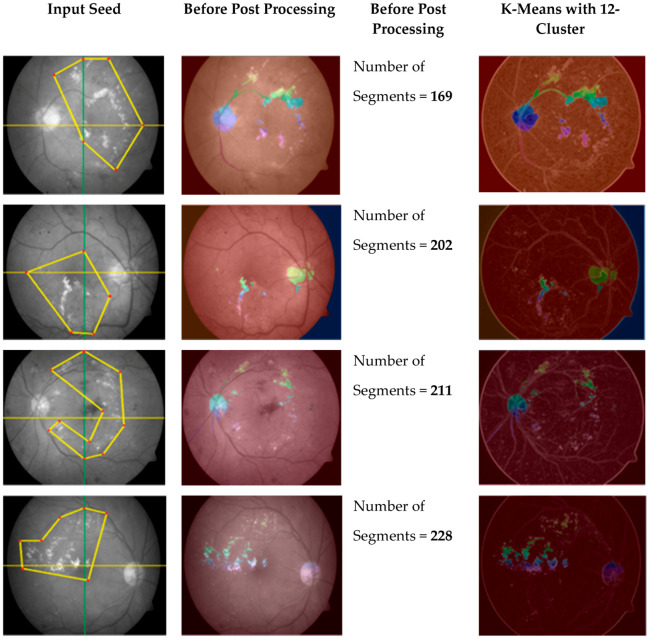
Irregular polygonal seed-based improved region growing outcome using K-mean clustering.

The new scheme avoids domain-specific problems, as fixed dimensions can be useless/not fit for all images, especially images with micro textures like bio medical images, where abnormalities are not observed in some regular shapes but can be seen when drawing irregular polygons with different dimensions. Thus, we can expect calculations to be more accurate because they cover the maximum amounts of interested/abnormal regions (in case of medical images) accordingly. The main advantage of this scheme over a scheme with regular polygons is the segmentation of a fewer possible and more specific homogeneous number of seeds with minimum noise, as shown in Figure 4.

By using the regular polygonal seed selection methodology for bio-medical, images like retinal fundus images slices are not connected, which results in extreme difficulty for useful segmentation [24]. However, an irregular polygonal seed selection scheme resolves this issue because it allows for any possible sizes, dimensions, and shapes to be considered as regions of interest (ROI). At the post processing stage, a K-mean algorithm [25,26] is employed on improved segmented regions, which results in the removal of noise for improved results.

Here, the RF image with an (i × j) resolution and RF image was divided into K number of clusters. The value of K is considered as a threshold point, the range is between 7 and 12. Let p (i × j) be the RF image seeds to cluster and K_C_ be the center point. Then, the K-mean clustering-based segmentation algorithm works as follows:

- First, set the K (threshold value) and center point.
- Second, calculate Euclidean distance *ED* for each seed of an RF image, between the center and each seed using the Equation (7):

(7) $$ED=\left|\left|\;p\left(i,\;j\right)-K_{C}\right|\right|$$

- The nearest cluster holds the seed based of distance.
- When all the seed have been allocated, recalculate the new location of center using Equation (8):

(8) $$K_{C}=\frac{1}{C}\;\underset{j\in K_{C}}{\sum }\underset{i\in K_{C}}{\sum }p\left(i,\;j\right)$$

- Continue the process unless it meets the value of tolerance or error.
- Reshape the cluster seeds into RF images.

**Figure 5:**
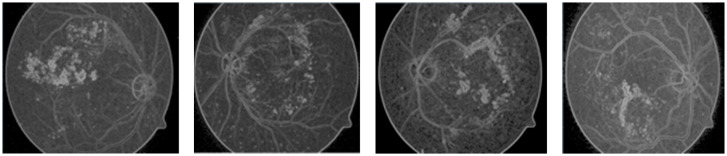
DR segmentation based on the watershed technique.

It has been experimentally proven that batch and volume processing for region growing are possible without expert knowledge, although it is a difficult process. The watershed (topographical) segmentation technique [27] that is used for retinal fundus image is not good enough because of issues like over-segmentation and sensitivity to noise; as a result of the watershed technique, any process is degraded by background noise and over-segmentation, as shown in Figure 5.

On the other hand, the proposed scheme uses marker-based segmentation approach radii, shapes, and centers of the irregular polygon-defined foreground, which improves segmentation results. The K-mean segmentation results are disturbed when K > 12 (K = 12; maximum threshold value) on retinal fundus images with the proposed seed-based segmentation scheme, which can successfully produce more useful clustering information compared to information produced by applying previous schemes, as shown in Figure 4.

### 2.4. Feature Extraction

For this study, a hybrid-feature dataset of DR was acquired using RF images; that is, the first-order histogram, second-order co-occurrence matrix, wavelet, and run-length matrix features. These features were grouped as 11 second-order co-occurrence matrix features including five average texture values in all four dimensions (0°, 45°, 90°, and 135°) and (11 × 5 × 4) a total of 220 calculated features, 15 run-length matrix features, 6 histogram features, and 4 wavelet features. Thus, 245 features per each ROI were extracted, and the total calculated features vector space (FVS) was 1,837,500 (245 × 7500) for the acquired RF image dataset. All these features were acquired using MaZda software, version 4.6. To carry out this study, all the experiments were carried out on an Intel^®^ Core i3 1.9 gigahertz (GHz) processor with 8 gigabytes (GB) of RAM and a 62-bit Windows 10 operating system.

#### 2.4.1. Run-Length Matrix (RLM)

Galloway [28] introduced the gray level run-length matrix (GLRM), a section of gray scale or color level—also known as a range or length of run—that is a linear multitude of continuous pixels with the same color or gray level in a particular direction. That is, $N_{g}$ is the total number of gray levels in the image, $N_{r}$ is the number of run lengths in the image, $N_{p}$ is the number of voxels in the image, N is the number of runs in the image in a particular direction, P(l,m) is the run length matrix for an particular direction, p(l,m)=P(l,m)/N is the normalized run length matrix for particular direction, and $u=\sum_{l=1}^{N_{g}}\sum_{m=1}^{N_{r}}i\;P\left(l,m\right)$. Then, the short run emphasis (SRE) is described in Equation (9):

(9) $$SRE=\frac{\sum_{l=1}^{N_{g}}\sum_{m=1}^{N_{r}}\frac{P\left(l,m\right)}{m^{2}}}{N}$$

The SRE measures the distribution of short runs. A high cost indicates a fine texture. Long run emphasis (LRE) measures the distribution of long runs. The high value indicates the coarse texture described in Equation (10):

(10) $$LRE=\frac{\sum_{l=1}^{N_{g}}\sum_{m=1}^{N_{r}}P\left(l,m\right)m^{2}}{N}$$

Gray level non-uniformity (GLN) measures run distribution over gray values. The cost of the feature is lower when the runs are evenly distributed. In this way, a lower value indicates a higher similarity in intensity values described in Equation (11):

(11) $$GLN=\frac{\sum_{l=1}^{N_{g}}{\left[\sum_{m=1}^{N_{r}}P\left(l,m\right)\right]}^{2}}{N}\;$$

Gray level non-uniformity normalized (GLNN) is a normalized version of the GLN feature defined in Equation (12):

(12) $$GLNN=\frac{\sum_{l=1}^{N_{g}}{\left[\sum_{m=1}^{N_{r}}P\left(l,m\right)\right]}^{2}}{N^{2}}$$

Run length non-uniformity (RLN) measures the distribution of runs over the run lengths. The cost of the feature decreases as the length of the run is evenly distributed, as defined in Equation (13):

(13) $$RLN=\frac{\sum_{m=1}^{N_{r}}{\left[\sum_{l=1}^{N_{g}}P\left(l,m\right)\right]}^{2}}{N}\;$$

Run length non-uniformity normalized (RLNN) is a normalized version of the RLN feature defined in Equation (14):

(14) $$RLNN=\frac{\sum_{m=1}^{N_{r}}{\left[\sum_{l=1}^{N_{g}}P\left(l,m\right)\right]}^{2}}{N^{2}}$$

Run percentage (RP) measures the fraction of the number of realized runs and the maximum number of potential runs. Strongly linear or highly uniform ROI volumes produce a low run percentage, as shown in Equation (15):

(15) $$RP=\frac{N}{N_{p}}\;$$

Low gray level run emphasis (LGLRE) is described in Equation (16):

(16) $$LGLRE=\frac{\sum_{l=1}^{N_{g}}\sum_{m=1}^{N_{r}}\;P\left(l,m\right)/l^{2}}{N}$$

High gray level run emphasis (HGLRE) is a grey level (GL) analogue to long run emphasis. The feature emphasizes high grey levels, as described in Equation (17):

(17) $$HGLRE=\frac{\sum_{l=1}^{N_{g}}\sum_{m=1}^{N_{r}}\;P\left(l,m\right)l^{2}}{N}$$

Short run low gray level emphasis (SRLGLE) emphasizes runs in the upper left quadrant of the GLRLM, where short run lengths (SRLs) and low GLs are located [29]. It is described in Equation (18):

(18) $$SRLGLE=\frac{\sum_{l=1}^{N_{g}}\sum_{m=1}^{N_{r}}\;P\left(l,m\right)/l^{2}m^{2}}{N}$$

Short run high gray level emphasis (SRHGLE) emphasizes runs in the lower left quadrant of the GLRLM, where SRLs and high GLs are located. It is given in Equation (19):

(19) $$SRHGLE=\frac{\sum_{l=1}^{N_{g}}\sum_{m=1}^{N_{r}}\;P\left(l,m\right)l^{2}/m^{2}}{N}$$

Long run low gray level emphasis (LRLGLE) emphasizes runs in the upper right quadrant of the GLRLM, where long run lengths (LRLs) and low GLs are located. It is defined in Equation (20):

(20) $$LRLGLE=\frac{\sum_{l=1}^{N_{g}}\sum_{m=1}^{N_{r}}\;P\left(l,m\right)m^{2}/l^{2}}{N}$$

Long run high gray level emphasis (LRHGLE) emphasizes runs in the lower right quadrant of the GLRLM, where LRLs and high GLs are located. It is presented in Equation (21):

(21) $$LRHGLE=\frac{\sum_{l=1}^{N_{g}}\sum_{m=1}^{N_{r}}\;p\left(l,m\right)l^{2}m^{2}}{N}$$

Grey level variance (GLV) measures the variance in runs for the GL. It is given in Equation (22):

(22) $$GLV=\overset{N_{g}}{\underset{l=1}{\sum }}\overset{N_{r}}{\underset{m=1}{\sum }}\;p\left(l,m\right){\left(l-u\right)}^{2}$$

Finally, run length variance (RLV) measures the variance in runs for run lengths. It is presented in Equation (23)

(23) $$RLV=\overset{N_{g}}{\underset{l=1}{\sum }}\overset{N_{r}}{\underset{m=1}{\sum }}\;p\left(l,m\right){\left(m-u\right)}^{2}$$

#### 2.4.2. Histogram Features (H)

Histogram features are used by selecting the object with respect to rows and column [30]. This binary object is used as a mask of the original image for feature extraction. Histogram features are calculated by the intensity of the individual pixels, which are the part of objects. These features are based on the histogram. They are also called first order histogram or statistical features. The first order histogram probability P(h) is described in Equation (24):

(24) $$P\left(h\right)=\frac{K\left(h\right)}{N}$$

It is clear that N represents the total numbers of pixels in the image and K(h) shows the complete instances of the gray scale value of h. The mean is average of the values; it describes the bright mean and dark mean in an image. The mean is defined as follows in Equation (25):

(25) $$\bar{i}=\overset{p-1}{\underset{h=0}{\sum }}hP\left(h\right)=\underset{m}{\sum }\underset{n}{\sum }\frac{k\;\left(m,n\right)}{k}$$

Here, q represents the grayscale values that range from 0 to 255. The consequent values of m (rows) and n (columns) show the pixel. Standard deviation (SD) describes the contrast of image. It is presented in Equation (26):

(26) $$\sigma_{h}=\sqrt{\overset{P-1}{\underset{h=0}{\sum }}{\left(h-\bar{h}\right)}^{2}P\left(h\right)}$$

The skewness measures a degree of asymmetry in comparison to a central value. It is defined as follows in Equation (27), and negative skewness is defined in Equation (28):

(27) $$Skewness=\frac{1}{\sigma_{h}^{3}}\overset{P-1}{\underset{h=0}{\sum }}{\left(h-\bar{h}\right)}^{3}\;P\left(h\right)$$

(28) $$-Skewness=\frac{\bar{h}-mode}{\sigma_{h}}$$

The gray level distribution is called energy. It is described in Equation (29):

(29) $$Energy=\overset{P-1}{\underset{h=0}{\sum }}{\left[P\left(h\right)\right]}^{2}\;$$

Entropy is described as randomness in the image data. It is defined in Equation (30):

(30) $$Entropy=-\overset{q-1}{\underset{i=0}{\sum }}q\;\left(i\right)log_{2}\left[q\left(i\right)\right]$$

#### 2.4.3. Co-Occurrence Matrix (COM)

COM (co-occurrence matrix) features are also called the second order statistical features. These features are obtained on the distance and the angle between pixels, based on gray level co-occurrence matrix (GLCOM) [31]. For this study, 11 second order texture features were calculated in four dimensions—0°, 45°, 90°, and 135°—up to a 5-pixel distance. If *P*_n_ is the normalized type of the gray level (g), the co-occurrence matrices of four directions like 0°, 45°, 90°, and 135° are described in Equation (31):

(31) $$P_{n}=\frac{1}{N_{c}}\left({\left[P\left(a,b\right)\right]}_{b=0,1,2,\ldots ,N_{g}-1}^{a=0,1,2,\ldots ,N_{g}-1}\right)$$

Here, the normalization constant $N_{c}$ can be expressed as in Equation (32):

(32) $$N_{c}=\overset{N_{g}-1}{\underset{a=0}{\sum }}\overset{N_{g}-1}{\underset{b=0}{\sum }}P\left(a,b\right)$$

First of all, energy is defined in Equation (33). Energy is calculated in distribution between gray level values:

(33) $$g_{E}=\overset{N_{g}-1}{\underset{a=0}{\sum }}\overset{N_{g}-1}{\underset{b=0}{\sum }}{\left\{P_{n}\left(a,b\right)\right\}}^{2}$$

The contrast is a difference moment of a matrix and it is a measure of local changing in the image. It is described in Equation (34):

(34) $$g_{c}=\overset{N_{g-1}}{\underset{n=0}{\sum }}n^{2}\left\{\overset{N_{g}-1}{\underset{a=0}{\sum }}\overset{N_{g}-1}{\underset{b=0}{\sum }}P_{n}\left(a,b\right)\right\}$$

Here, |a−b|=n. Correlation describes the pixel similarity at particular pixel distance. It is described in Equation (35):

(35) $$g_{cor}=\frac{\sum_{a=0}^{N_{g}-1}\sum_{b=0}^{N_{g}-1}\left(rc\right)P_{n}\left(a,b\right)-\mu_{H}\mu_{V}}{\sigma_{H}\sigma_{V}}$$

where $\mu_{H}$, $\mu_{V}$, $\sigma_{H}$, and $\sigma_{V}$ are the means and standard deviations of the marginal probability matrices *MH* and *MV*, respectively and normalized gray level denoted by Ng, which are shown as $M_{H}={\left[M_{H}\left(a\right)\right]}_{b=0,1,2,\ldots ,N_{g}-1}$ and $M_{V}={\left[M_{V}\left(b\right)\right]}_{a=0,1,2,\ldots ,N_{g}-1}$. Moreover, $M_{H}\left(a\right)=\overset{N_{g}-1}{\underset{b=0}{\sum }}M_{n}\left(a,b\right)$, a= 0, 1, 2,…, *Ng* – 1. While, *a* remains constant in complete row, the column sum of *Mn.* Similarly, $M_{V}\left(b\right)=\overset{N_{g}-1}{\underset{a=0}{\sum }}M_{n}\left(a,b\right)$, b = 0, 1, 2,…, *Ng* − 1. Additionally, *b* is constant in whole column, which means that is the row sum of *Mn*.

Additionally, $M_{H+V}={\left[M_{H+V}\left(k\right)\right]}_{k=0,1.2,3,\ldots ,2N_{g}-2}$, $M_{H+V}\left(k\right)=\overset{N_{g}-1}{\underset{a=0}{\sum }}\overset{N_{g}-1}{\underset{b=0}{\sum }}M_{n}\left(a,b\right)$, where k = a + b, $M_{H-V}={\left[M_{H-V}\left(k\right)\right]}_{k=0,1.2,3,\ldots ,N_{g}-1}$, $M_{H-V}\left(k\right)=\overset{N_{g}-1}{\underset{a=0}{\sum }}\overset{N_{g}-1}{\underset{b=0}{\sum }}M_{n}\left(a,b\right)$, and k = |a − b|.

This feature gives a measure of variability in specific regions of an image. If gray values have low differences in an image, then variance is low. This is described in Equation (36):

(36) $$g_{var}=\overset{N_{g}-1}{\underset{a=0}{\sum }}\overset{N_{g}-1}{\underset{b=0}{\sum }}{\left(a-\mu \right)}^{2}M_{n}\left(a,b\right)$$

The local homogeneity of the image is called the inverse difference, and it is presented in Equation (37):

(37) $$g_{IDM}=\overset{N_{g}-1}{\underset{a=0}{\sum }}\overset{N_{g}-1}{\underset{b=0}{\sum }}\frac{1}{1+k^{2}}M_{n}\left(a,b\right)$$

Difference variance is defined in Equation (38):

(38) $$g_{DV}=\overset{N_{g}-1}{\underset{a=0}{\sum }}{\left(a-\mu \right)}^{2}M_{x-y}\left(v\right)\;$$

Sum average is presented in Equation (39):

(39) $$g_{SA}=\overset{2N_{g}-2}{\underset{a=0}{\sum }}aM_{x+y}\left(a\right)$$

Sum variance is described in Equation (40):

(40) $$g_{SV}=\overset{2N_{g}-2}{\underset{v=0}{\sum }}{\left(a-\mu \right)}^{2}M_{x+y}\left(a\right)$$

Entropy measures the total content of the image. It is described in Equation (41):

(41) $$g_{EN}=-\overset{N_{g}-1}{\underset{a=0}{\sum }}\overset{N_{g}-1}{\underset{b=0}{\sum }}M_{n}\left(a,b\right)log\left(M_{n}\left(a,b\right)\right)$$

Sum entropy is presented in Equation (42):

(42) $$g_{SEN}=-\overset{2N_{g}-2}{\underset{a=0}{\sum }}M_{x+y}\left(a\right)log\left\{M_{x+y}\left(a\right)\right\}$$

Finally, difference entropy is defined in Equation (43):

(43) $$g_{DEN}=\overset{N_{g}-1}{\underset{a=0}{\sum }}M_{x-y}\left(a\right)log\left\{M_{x-y}\left(s\right)\right\}$$

#### 2.4.4. Wavelet-Based Features (W)

Generally, discrete wavelet transforms (DWT) features are the linear transformation which implemented on a data vector whose value is an integer power of two, changing it numerically different data vectors in the same quantitative values. It means data is segmented into different frequency resolutions. After this DWT value is calculated by using successive flow by using a factor of two subsampling which is high pass filters (H) and low pass filters (L). For this study the Harr wavelet feature are calculated for DR dataset. This dataset is segmented into four sub bands (HH, HL, LH, and LL) at each scale. Sub band X^LL^ is used to compute the DWT features. For DR dataset, the Harr wavelet features computed only if output sub bands have at least 8 by 8 dimension. Furthermore, the Equation (44) described the DWT features calculated on a given ROIs [32]:

(44) $$Energy_{Sub-band,\;scale}=\frac{\sum_{y,z\in RigionOfIntrest}{\left(d_{y,\;z}^{sub-band}\right)}^{2}}{k}$$

where the constraint $d_{y,z}$ is the resultant matrix component. Σ is performed for each pixel (y, z) situated in the region of interest definition, and K is the total number of pixels in ROIs.

### 2.5. Feature Selection

Feature selection is the most important part of the ML process. The main goal of this process is to select the most worthwhile features and remove the most worthless features in a dataset. This process deals with the elimination of missing values and highly correlated, low variance, and recursive features, and it also deals with univariate feature selection by using ML. Usually, an acquired dataset is a combination of large numbers of features that are difficult to handle. It is essential to reduce the dimensionality of this feature vector space, which can efficiently distinguish and classify the different classes. These techniques can be implemented to achieve the most discriminating features and to get cost-effective classification results [33].

In this study, it was observed that all the extracted features were not equally worthwhile for DR classification. Since the acquired dataset had a large FVS (precisely 1,837,500), it was very difficult to deal with. To solve this problem, three ML-based feature selection techniques, namely F, MI, and probability of error (POE) plus average correlation (AC), were used to select the optimize features for the acquired DR dataset. This approach was the combination set of above mention ML techniques (F, MI, and PA) [34] that selected 30 pre-optimized features out of 245 features using the MaZda software [35]. Mathematically, the F technique can be represented as:

(45) $$F=\;\frac{N^{2}}{V^{2}}=\frac{\left[1/\left(1-\sum_{i=1}^{i}\;\rho_{i}^{2}\right)\right]\sum_{i=1}^{i}.\;\sum_{y=1}^{i}\rho_{i\;}\;\rho_{y}{\left|L_{i}-L_{y}\right|}^{2}}{\sum_{i=1}^{i}.\;\rho_{i}M_{i}^{2}}$$

where N is between-class variance, V is within-class variance, $\rho_{i}$ is probability of feature i, and $M_{i}$ and $L_{i}$ are the variance and mean value of feature i in the given class, respectively. In addition, POE and AC are defined as:

(46) $$PA\left(f_{y}\right)=\frac{MissclassifiedSamples}{TotalSamples}$$

(47) $$f_{2}=f_{y}\;:\;Min_{y}\left[\;PA\left(f_{y}\right)+\left|Correlate\;\left(f_{1},\;f_{y}\right)\right|\right]$$

(48) $$f_{k}=f_{y}\;:\;Min_{y}\left[\;PA\left(f_{y}\right)+\frac{1}{N-1}\;\left|Correlate\;\left(f_{1},\;f_{y}\right)\right|\right]$$

Additionally, MI is represented as:

(49) $$MI\left(I,\;J\right)=\sum^{\;}I\sum^{\;}J.\;\;P\left(I.J\right)Log^{2}P\;\frac{P\left(I.J\right)}{P\left(I\right).P\left(J\right)}$$

In this study, it was observed that the above feature selection (F, MI, and PA) techniques had some limitations. They selected exactly 30 features, but only a few of them were worthwhile. Thus, one more feature selection technique, namely CFS, was used on pre-an optimized dataset [36]. CFS can extract the most projecting features in a dataset. CFS uses the information theory based on the entropy presented in Equation (50):

(50) $$H\left(Y\right)=-\sum^{\;}P\left(y_{i}\right)log2\;P\left(y_{i}\right)$$

The entropy of Z after observing values of another variable W is defined in Equation (51):

(51) H(Y|X) =−∑ P(xj)∑ P(yixj)log2 P(yi)

Here, *P* $\left(y_{i}\right)$ the primary probabilities for all values of *Y* and *P* (yi/xj) is the secondary probabilities of *Y* when values of *X* are given. The value found when the entropy of *Y* is reduced shows more information about *Y* given by *X*, and this is called additional information; see Equation (52):

(52) IA(Y|X)=T(Y)−T(Y|X)

Here, the *X* feature is more correlated to the *Y* feature than to the *Z* feature if the following inequality holds:

(53) IA(Y|X)>IA(Z|X)

We have to quantify symmetrical uncertainty (*SYU*), which expresses the correlation among features. It is described in Equation (54):

(54) SYU(Y,X)=2{IA(Y|X)|T(Y)+T(X)}

Entropy-based quantification needs normal features, but it is implemented to properly calculate the correlations between continuous and discretized features. CFS was deployed on the pre-optimized dataset that selected 13 post-optimized features for further processing. The post-optimized features are shown in Table 1.

**Figure 6:**
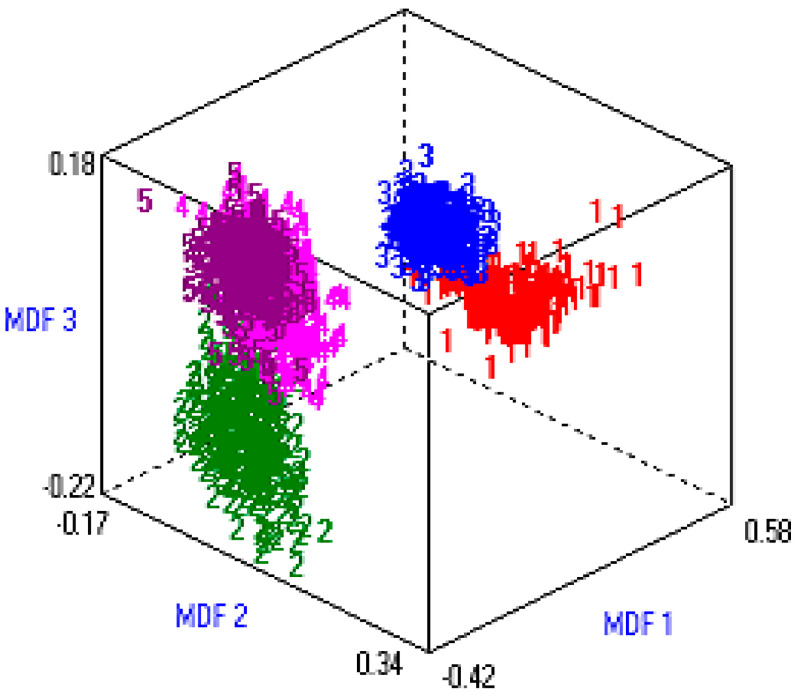
Data visualization in 3D vector space.

Finally, the 1,837,500 (245 × 7500) fused hybrid-features vector space was reduced to 97,500 (13 × 7500). CFS-based post-optimized datasets were generated for each type of extracted feature for DR and these post-optimized, fused hybrid-feature datasets were deployed to different machine learning classifiers. Figure 6 shows the three-dimensional (3D) representation of the post-optimized features. MDF1, MDF2, and MDF3 are three different dimensions (like x, y, z) of most discriminant features.

### 2.6. Classification

Five ML classifiers—SMO, Lg, MLP, LMT, and SLg—were employed on RF image datasets. First, it was observed that by employing these classifiers on the histogram, wavelet, and co-occurrence matrix feature datasets, unfortunately, a very low accuracy was found, with values of 73.73%, 79.93% and 89.93%, respectively. Similarly, when the same classifiers with the same strategies were deployed on the run-length matrix features, the overall accuracy of 95.93% was observed, which was slightly better than that of the other feature’s datasets. Finally, to obtain a more promising accuracy, we generated a post-optimized, fused hybrid-feature dataset, and very impressive results were observed for the deployed classifiers. Here, the SLg classifiers performed the best among the implemented classifiers, though it mostly performed well for noisy, big, and complex data [35]. The SLg classifier is theoretically expressed in Equation (55):

(55) $$log\left(\frac{P}{1-P}\right)=w_{0}+w_{1}x_{1}+w_{2}x_{2}+\ldots +w_{k}x_{k}$$

where:

(56) $$p=\frac{1}{1+b^{-\left(w_{0}+w_{1}x_{1}+w_{2}x_{2}+\ldots +w_{k}x_{k}\right)}}$$

and x_1_ … x_k_ are the explanatory variables with parameters w_0_, w_1_,…, w_k_.

## 3. Results and Discussion

For this study, five ML classifiers named SMO, Lg, MLP, LMT, and SLg were employed on selected post-optimized hybrid feature datasets (using 10-fold cross-validation) for the classification of DR. As discussed earlier, four types of texture features, namely H, W, COM, and RLM, were extracted using the RF image datasets. In the first step, the histogram features-based dataset was used for DR classification, and this did not give better results than the other employed classifiers, at less than or equal to 73.73%. In the second step, the same approach was employed on the wavelet features-based dataset, where the overall classification accuracy of the employed classifiers was less than or equal to 79.93%. Here, we observed some improvement in accuracy compared to the previous results, but it was not so inspiring. In the third step, the same approach was employed on the co-occurrence matrix features-based dataset, and it was observed that the overall classification accuracy of the employed classifiers was less than or equal to 89.93%. This was a very promising improvement in accuracy compared to the previous results, but this process will continue for further analysis. In the fourth step, the same approach was employed on run-length matrix features-based dataset, and the overall classification accuracy of the employed classifiers was less than or equal to 95.93%; we observed a very promising improvement in accuracy compared to the previous results. In this analysis, we observe that each texture feature carried its worth in the DR analysis case, and the most worthwhile feature was a run-length feature that gave the highest classification accuracy compared to others. To improve classification accuracy, a post-optimized, fused hybrid-feature dataset was generated by applying the data fusion approach, which is a very powerful technique for merging multiple features to produce an accurate classification compared to individual features. In the last step, the same approach was employed on a post-optimized, fused hybrid-features dataset, and the employed classifiers, SMO, Lg, MLP, LMT, and SLg showed very high classification accuracies of 98.53%, 99%, 99.66%, 99.73%, and 99.73%, respectively. The overall classification results of the histogram features-based dataset with the employed ML classifiers with other performance evaluating factors such as kappa statistics, true positive (TP), false positive (FP), ROC, mean absolute error (MAE), root mean squared error (RMSE), confusion matrix, time complexity (T), and overall accuracy (OA) are shown in Table 2.

**Figure 7:**
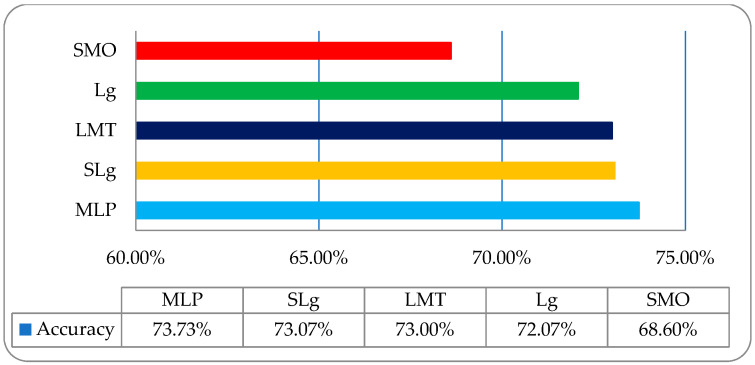
The overall accuracy graph of the employed ML classifiers on a histogram features-based dataset.

It was observed that in the employed classifiers on histogram features, the MLP classifier showed a relatively better classification accuracy of 73.73% compared to other employed classifiers, as shown in Figure 7. To acquire some improvement in classification accuracy, we then employed the same classifiers on a wavelet features-based dataset, as shown in Table 3.

**Figure 8:**
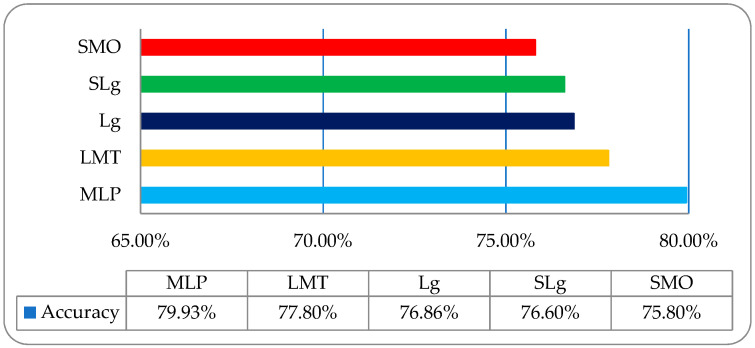
The overall accuracy graph of the employed ML classifiers on a wavelet features-based dataset.

It was observed that the overall accuracies of MLP, LMT, Lg, SLg and SMO were 79.93%, 77.80%, 79.86%, 76.60%, and 75.80%, respectively, as shown in Figure 8.

**Figure 9:**
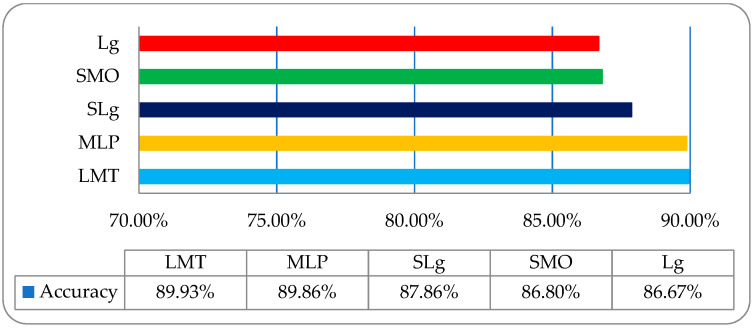
The overall accuracy graph of the employed ML classifiers on a COM features-based dataset.

To acquire some more improvement in classification accuracy, we then employed the same classifiers on a co-occurrence matrix (COM) features-based dataset, as shown in Table 4. It was observed that was the overall accuracies of LMT, MLP, SLg, SMO, and Lg were 89.93%, 89.86%, 87.86%, 86.80%, and 86.67%, respectively, as shown in Figure 9.

To acquire some more promising improvement in classification accuracy, we then employed the same classifiers on a run-length matrix (RLM) features-based dataset, as shown in Table 5.

**Figure 10:**
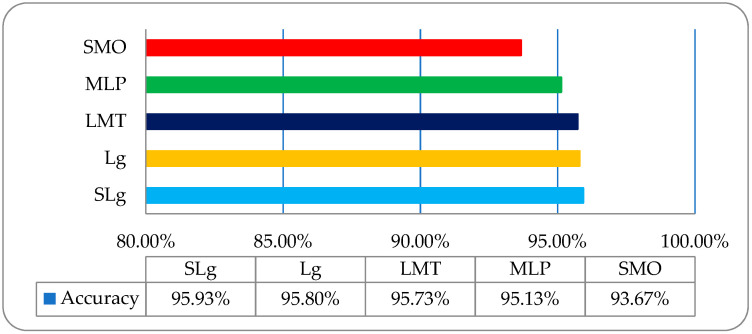
The overall accuracy graph of the employed ML classifiers on an RLM features-based dataset.

It was observed that the overall accuracies of SLg, Lg, LMT, MLP, and SMO were 95.93%, 95.80%, 95.73%, 95.13%, and 93.67%, respectively, as shown in Figure 10.

**Figure 11:**
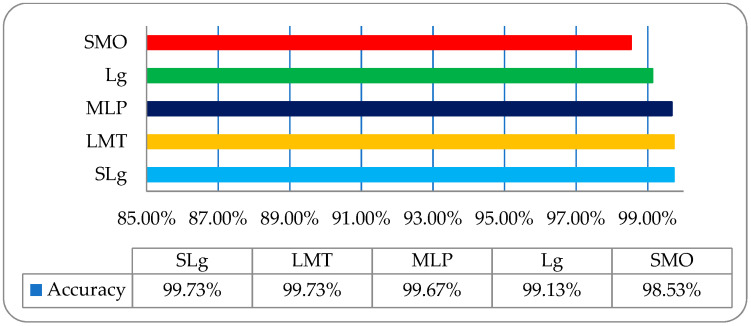
The overall accuracy graph of the employed ML classifiers on a post-optimized fused dataset.

Finally, the overall accuracy of the histogram, wavelet, COM, and RLM features were not so impressive; meanwhile, on a post-optimized, fused hybrid-features-based dataset, the same strategy with the same classifiers was employed, and very promising results were observed. The overall classification accuracies of the employed classifiers of SLg, LMT, MLP, Lg, and SMO were 99.73%, 99.73%, 99.67%, 99.13%, and 98.53% respectively, as shown in Figure 11. These results were very inspiring, and it was observed that SLg and LMT showed the same accuracy; however, when including the time factor, the SLg showed the best accuracy among all the implemented classifiers. The overall accuracy of SLg with others implemented ML classifiers on a post-optimized, fused hybrid-feature dataset with performance evaluating parameters is shown in Table 6.

Similarly, the confusion matrix (CM) of the post-optimized, fused hybrid-features dataset is shown in Table 7. The colored diagonal of the CM (Table 7) shows the classification accuracy in appropriate classes, while other instances show them in other classes. This table contains the information that is actual and predicted data for the SLg classifier. The SLg classifier showed a relatively better overall accuracy than any of the implemented classifiers.

**Figure 12:**
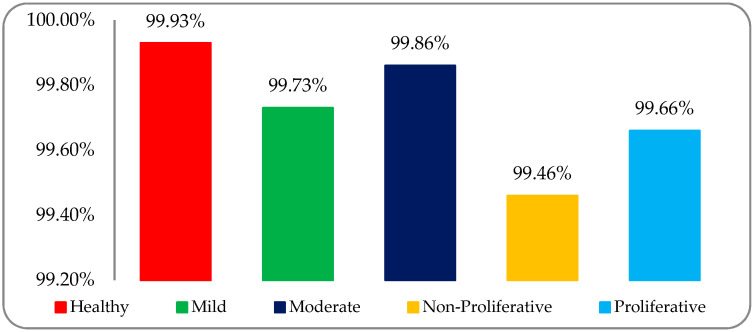
Classification accuracy graph of five DR stages using the SLg classifier on a post-optimized fused dataset.

The classification accuracy results of the five DR stage (both healthy and DR) RF datasets—that is, healthy, mild, moderate, non-proliferative, and proliferative—were 99.93%, 99.73%, 99.86%, 99.46%, and 99.66%, respectively. The graphical accuracy results are shown in Figure 12.

**Figure 13:**
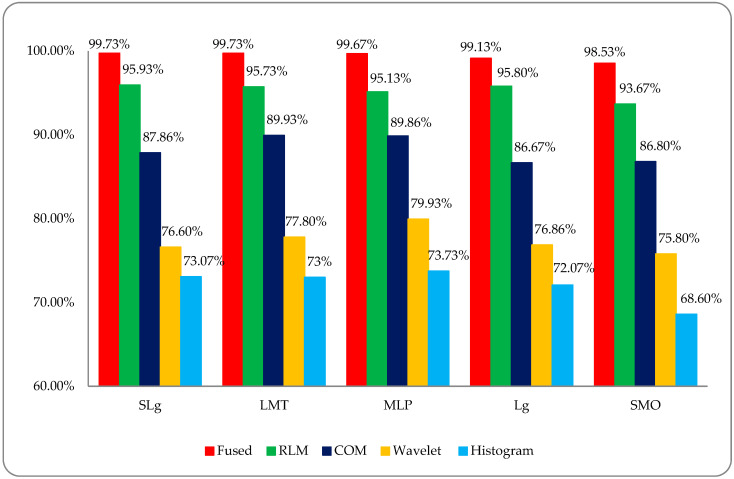
Comparative analysis of DR stage classification accuracies among different type of feature modalities.

Finally, a comparative DR classification graph was made of the histogram, wavelet, co-occurrence matrix (COM), run-length matrix (RLM), and post-optimized, fused hybrid-feature datasets using the employed ML classifiers; this is shown in Figure 13. This graph shows the overall better accuracy (RED) for the DR classification that was found by using a post-optimized, fused hybrid-feature dataset compared to RLM (GREEN), COM (BLUE), wavelet (YELLOW), and histogram (SKY-BLUE) features-based datasets.

DR is caused by damage to the blood vessels of the light-sensitive tissue at the back of the eye (retina). Due to this damage, the fine texture of the retina is disturbed, and, due to this problem, texture features play an important role in DR classification. After reviewing the literature, we found different kinds of research work on different types of texture features. In this study, a comparative analysis was performed between all of these features and a fused hybrid feature dataset, which was the combination of all these features (as shown in Figure 13). The existing system has some limitations because it is a supervised, learning-based classifier. All testing was performed in a retina fundus image dataset that was acquired to meet all legal requirements of the ophthalmology department of the BVH, Bahawalpur, Pakistan [20], with the collaboration of the Department of Computer Science of the Islamic University from Bahawalpur (IUB) [37], Pakistan, to address the regional and local problem of identifying diabetic retinopathy stages. The proposed model was verified on the publicly available datasets of the diabetic retinopathy (DR) archives the High-Resolution Fundus (HRF) Image and Messidor-2 (M2) [38,39] databases. Fifty patients’ data of five stages of DR were acquired from the HRF and M2 datasets using a Canon CR-1 fundus camera with a field of view of 45° and different acquisition settings. Twenty retinal fundus images of each stage and total of 100 (20 × 5) datasets of five DR stages (where one is a normal patient retina and the other four are DR patient stages, namely mild, moderate, non-proliferative, and proliferative) were acquired from these public archives. The multi-institutional dataset differences between HRF, M2, and BVH were observed, and we tried our best to normalize the HRF and M2 datasets with respect to the BVH dataset. At first, we resized each slice of the HRF and M2 datasets as per the standard of BVH, and then we employed the proposed technique (CARGS) with the same classifiers shown in Table 8.

**Figure 14:**
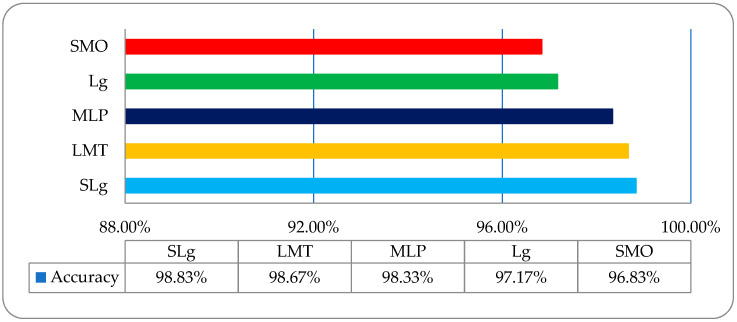
The overall accuracy graph of the employed ML classifiers on post-optimized, fused public dataset.

A very promising result was observed with a variation of 96.8333–98.8333% of classification accuracy, as shown in Figure 14.

**Figure 15:**
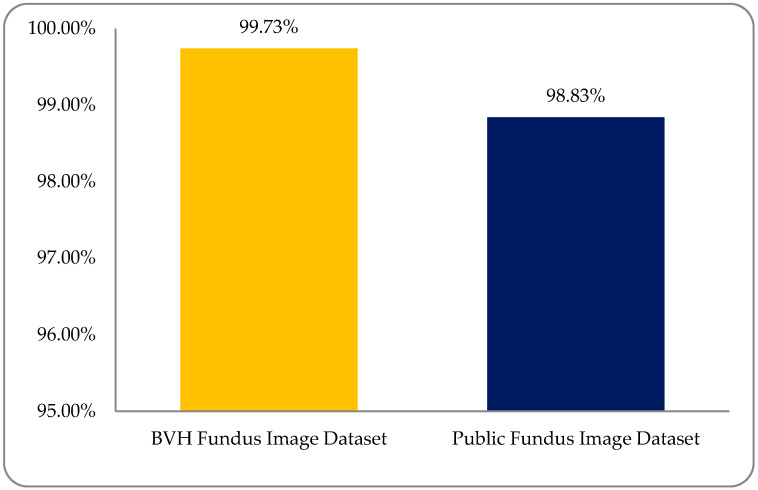
A comparison accuracy graph between Bahawal Victoria Hospital (BVH) and public (RF image) dataset with the SLg classifier.

Experimental results were observed to vary due to variations in the multi-institutional RF image datasets. We must support the development of a dataset of the global platform for medical patient data in which—despite differences in patient mode, region, demography, geography, and medical history—we can more accurately address these medical health issues. A comparison graph between the BVH and public RF image datasets is shown in Figure 15.

A comparison between the proposed methodology and the current state-of-the-art techniques in is Table 9.

## 4. Conclusions

This study focused on the classification of four DR stages (mild, moderate, non-proliferative, and proliferative), as well as the normal human retina, with the help of fused hybrid-feature analysis using fundus images by applying ML classifiers. The main goal was K-mean clustering-based segmentation, the selection of appropriately optimized hybrid-features, and the identification of suitable classifiers for well-organized classification. The variation of results was due to different modalities of texture analysis. Four types of features were extracted, namely histogram, wavelet, co-occurrence matrix, and run-length matrix. In the end, a fused hybrid-feature dataset, which is a combination of the above-mentioned extracted features, was generated. The fused hybrid-feature dataset was generated by using a data fusion approach. A large number of features can minimize the accuracy of classification and maximize total execution time, so the main focus was applied to the feature optimization process and used with a post-optimized hybrid-feature dataset. This study concluded that more accurate and precise result can be achieved by applying five ML classifiers—SLg, LMT, MLP, Lg, and SMO—on post-optimized hybrid-feature datasets. All the deployed classifiers showed promising results, but the SLg classifier results were outstandingly high. After employing the SLg classifier, it was observed that an overall accuracy of 99.73% was achieved. The accuracies obtained by SLg on the five DR classes of healthy, mild, moderate, non-proliferative, and proliferative were 99.93%, 99.73%, 99.86%, 99.46%, and 99.66%, respectively. The proposed model was verified on publicly available, high-resolution fundus, and Messidor-2 datasets. A very promising result was observed with a variation of 96.83–98.83% of classification accuracy.

### Future Work

In the future, this study will assist in building up an image dynamic searching approach with deep learning algorithms to increase the momentum for classification. This technique can be further improved by using the 3D visualization of volumetric RF data. Among the planed future works, clinical applicability will be observed for this proposed model.

## Figures and Tables

**Table 1:** Post-optimize feature selection table (Fisher (F), probability of error (POE) plus average correlation (AC), mutual information (MI), and correlation-based feature selection (CFS)).

**Features**	***Run Length***	***Co-Occurrence***	***Wavelet***	***Histogram***
	1. 45dgr_RLNonUni 2. 135dr_RLNonUni 3. Horzl_RLNonUni 4. Vertl_RLNonUni 5. 45dgr_GLevNonU	6. S(5,0)Entropy 7. S(5,5)Contrast 8. S(5,-5)Contrast	9. WavEnHL_s-4 10. WavEnLL_s-4	11. Variance 12. Skewness 13. Perc.01%

**Table 2:** The overall classification accuracy table of the employed machine learning (ML) classifiers on a histogram features-based dataset. ROC: receiver-operating characteristic; TP: true positive; FR: false positive; MAE: mean absolute errors; RMSE: root mean squared error; OA: overall accuracy; MLP: multi-layer perceptron; SLg: simple logistic; LMT: logistic model tree; Lg: logistic; and SMO: sequential minimal optimization.

Classifiers	Kappa Statistics	TP Rate	FP Rate	ROC	MAE	RMSE	Time (s)	OA
MLP	0.6717	0.737	0.066	0.916	0.1485	0.2799	0.78	73.73%
SLg	0.6633	0.731	0.067	0.921	0.1637	0.2813	0.59	73.07%
LMT	0.6625	0.730	0.068	0.919	0.1534	0.2807	0.52	73.00%
Lg	0.6617	0.729	0.068	0.923	0.1556	0.2789	0.37	72.07%
SMO	0.6075	0.686	0.09	0.878	0.2602	0.3465	0.25	68.60%

**Table 3:** The overall classification accuracy table of the employed ML classifiers on a wavelet features-based dataset.

Classifiers	Kappa Statistics	TP Rate	FP Rate	ROC	MAE	RMSE	Time (s)	OA
MLP	0.7492	0.799	0.050	0.943	0.0894	0.2559	1.02	79.93%
LMT	0.7225	0.778	0.056	0.933	0.1072	0.2571	1.98	77.80%
Lg	0.7108	0.769	0.058	0.944	0.1299	0.2581	0.04	76.86%
SLg	0.7075	0.766	0.059	0.945	0.1362	0.2582	0.98	76.60%
SMO	0.6975	0.758	0.061	0.914	0.2533	0.3367	0.09	75.80%

**Table 4:** The overall classification accuracy table of the employed ML classifiers on co-occurrence matrix (COM) features-based dataset.

Classifiers	Kappa Statistics	TP Rate	FP Rate	ROC	MAE	RMSE	Time (s)	OA
LMT	0.8742	0.899	0.025	0.982	0.057	0.1755	0.99	89.93%
MLP	0.8733	0.899	0.025	0.891	0.046	0.1865	1.21	89.86%
SLg	0.8483	0.879	0.030	0.981	0.075	0.1886	0.43	87.86%
SMO	0.835	0.868	0.033	0.950	0.247	0.3269	0.12	86.80%
Lg	0.8583	0.887	0.028	0.978	0.051	0.1896	0.09	86.67%

**Table 5:** The overall classification accuracy table of the employed ML classifiers on a run-length matrix (RLM) features-based dataset.

Classifiers	Kappa Statistics	TP Rate	FP Rate	ROC	MAE	RMSE	Time (s)	OA
SLg	0.9492	0.959	0.010	0.996	0.0307	0.1167	0.62	95.93%
Lg	0.9475	0.958	0.011	0.996	0.0247	0.1166	0.72	95.80%
LMT	0.9467	0.957	0.011	0.994	0.0292	0.1186	0.82	95.73%
MLP	0.9392	0.951	0.012	0.994	0.0292	0.1242	0.98	95.13%
SMO	0.9208	0.937	0.016	0.978	0.243	0.3209	0.06	93.67%

**Table 6:** The overall classification accuracy table of the employed ML classifiers on a post-optimized, fused hybrid-features dataset.

Classifiers	Kappa Statistics	TP Rate	FP Rate	ROC	MAE	RMSE	Time (s)	OA
SLg	0.9967	0.997	0.001	1.000	0.0051	0.0367	0.32	99.73%
LMT	0.9967	0.997	0.001	1.000	0.0048	0.0353	0.58	99.73%
MLP	0.9958	0.997	0.001	1.000	0.0038	0.0323	0.33	99.67%
Lg	0.9892	0.991	0.002	0.999	0.0036	0.0566	0.42	99.13%
SMO	0.9817	0.985	0.004	0.996	0.2406	0.3172	0.21	98.53%

**Table 7:** The confusion matrix of post-optimized, fused hybrid-feature dataset for the SLg classifier.

Classified as	Healthy	Mild	Moderate	Non-Proliferative	Proliferative	Total
**Healthy**	**1499**	0	0	0	0	**1500**
**Mild**	0	**1496**	0	0	0	**1500**
**Moderate**	1	0	**1498**	0	0	**1500**
**Non-Proliferative**	0	0	0	**1492**	1	**1500**
**Proliferative**	0	1	0	1	**1495**	**1500**

**Table 8:** The overall classification accuracy table of the employed ML classifiers on a post-optimized, fused public dataset.

Classifiers	Kappa Statistics	TP Rate	FP Rate	ROC	MAE	RMSE	Time (s)	OA
SLg	0.9767	0.988	0.012	1.000	0.0191	0.0914	0.38	98.83%
LMT	0.9733	0.987	0.013	0.985	0.0165	0.1137	0.18	98.67%
MLP	0.9667	0.983	0.017	0.998	0.0268	0.1089	0.28	98.33%
Lg	0.9433	0.972	0.028	0.998	0.0313	0.1404	0.39	97.17%
SMO	0.9367	0.968	0.032	0.997	0.0302	0.1542	0.11	96.83%

**Table 9:** A comparison table among the proposed and current state-of-the-art techniques.

Source/Reference	Methodology	Modality	Accuracy
Pires, R. et al. [10]	Convolutional Neural Networks	RF Image	99.0%
Zhang, W. et al. [11]	Neural Networks	RF Image	98.1%
Harun, N. H. et al. [12]	MLP and Artificial Neural Network	RF Image	72.11%
Verbraak, F. D. et al. [13]	Hybrid Features and Deep Learning	RF Image	93.8%
Afrin, R. and Shill, P. C. [14]	Fused Feature and Fuzzy Logic	RF Image	95.63%
Parmar, R. et al. [15]	Neural Networks	RF Image	85%
Xu, K. et al. [16]	Neural Networks	RF Image	94.5%
Gulshan, V. et al. [18]	Deep Learning	RF Image	97.5%
Gargeya R. [19]	Data-Driven Deep Learning Algorithm	RF Image	94%
**Proposed Methodology**	CARGS, Post-Optimized, Fused Hybrid-Features, and Simple Logistic	BVH RF Image Dataset	99.73%
**Proposed Methodology** **(Validation)**	CARGS, Post-Optimized, Fused Hybrid-Features, and Simple Logistic	Publicly Available Dataset (RF-Image)	98.83%
